# Unroofing and curettage for recurrent sacrococcygeal pilonidal disease

**DOI:** 10.3389/fsurg.2024.1456846

**Published:** 2024-11-20

**Authors:** Mehmet Ali Koc, Haydar Celasin, Kaan Sunter, Cihangir Akyol, Ibrahim Ethem Gecim

**Affiliations:** ^1^ Department of Surgery, Ankara University School of Medicine, Ankara, Türkiye; ^2^ Department of Surgery, Lokman Hekim University School of Medicine, Ankara, Türkiye

**Keywords:** sacrococcygeal pilonidal disease, recurrence, unroofing, curettage, pilonidal cyst, pilonidal sinus

## Abstract

**Purpose:**

Sacrococcygeal pilonidal disease (SPD) is a global concern, notably in Southeast Europe and the Middle East. Unroofing curettage (UC), which provides faster recovery, better cosmetic appearance, and lower recurrence rates in the primary cases, was evaluated with the results of recurrent disease.

**Methods:**

This retrospective study included 74 patients with recurrent disease who were over 16 years of age, experienced recurrence after at least one surgical attempt, and underwent unroofing curettage between 2007 and 2019. Operation time, return to work duration, and recurrence rates were assessed.

**Results:**

Mean age of patients was 29.8 ± 10.6 years, and 61 (82.4%) were male. Previous procedures included excision + flap reconstruction, excision + primary closure, and local excision + lay open. Mean operation time for unroofing curettage was 22 ± 5.3 min. Mean durations for return to work and recovery were 5.9 ± 3 days, and 6.5 ± 2.6 weeks, respectively. Mean follow-up duration was 81.6 ± 49 months. Recurrence was only observed in 1 (1.3%) patient. Unroofing curettage showed a mean recurrence-free period of 156.9 months (95% CI [, 152.9–160.9 months).

**Conclusion:**

Unroofing curettage stands out as a low-recurrence approach, likely to persist as a treatment method, especially for a selected group with recurrence.

## Introduction

Sacrococcygeal pilonidal disease (SPD) is a common condition, especially in young men, impairing quality of life. The incidence of the disease, preferred treatment methods and recurrence rates vary geographically (1). Globally, the incidence of SPD is approximately 1/1,000, and in Turkey, it is even higher (1). For the treatment of SPD, the need for a method with a shorter healing time, better cosmetic results, and lower recurrence rate has been discussed since the 19th century (2). A wide variety of treatment methods have been proposed, and none are perfect. All of them have specific advantages and disadvantages. While recurrence rates are reported to be low with local excision, this technique undoubtedly has the longest recovery time (3). Although comparable recurrence rates have also been reported with flap methods, cosmetic outcomes are relatively poor (4). Minimally invasive methods such as phenol or laser ablations and endoscopic pilonidal sinus treatment (EPSiT) or their combinations have very short recovery times. Laser therapy and EPSiT have faced criticism; however, their advantages, such as faster return-to-work times, are anticipated to receive further attention (2–5). In contrast, unroofing curettage (UC) has emerged as a primary method due to its low recurrence rates, early return to work, short operation time, and relatively better cosmetic outcomes, particularly in primary cases (5–7).

The preferred treatment method for recurrent pilonidal sinus cases is another subject of debate. As unsuccessful treatment may turn a simple disease into a stubborn chronic health problem in some patients, preventing recurrence and morbidity should probably be the most important criterion when selecting the surgical technique. In this study, we investigated the effectiveness of the UC technique for cases of recurrent SPD.

## Methods

### Study design and population

The study was approved by a local Institutional Review Board (No. i02-129-24). This retrospective study was conducted in three different institutions to examine the UC procedure for recurrent pilonidal disease performed by a single team. Patients who underwent UC for pilonidal disease between 2007 and 2019 were included. Patients who were older than 16 years of age and with active disease that recurred after at least 1 surgical attempt were included. Patients under the age of 16 and cases with no previous surgical attempt were excluded.

### Preoperative preparation and surgical technique

All procedures were performed in the prone position. All patients received intravenous sedation using midazolam (Dormicum®, Roche) 0.02–0.03/kg and propofol 1–2 mg/kg. (Propofol, Fresenius) and were monitored under supervision of an anesthetist. A maximum volume of 40 ml of prilocaine 1% solution was used for local anesthesia. The skin was shaved in the operation room immediately before surgery. The gluteal skin was taped and pulled outward bilaterally for better vision of the surgical site. The surgical field was prepped with povidone iodine solution.

**Figure 1:**
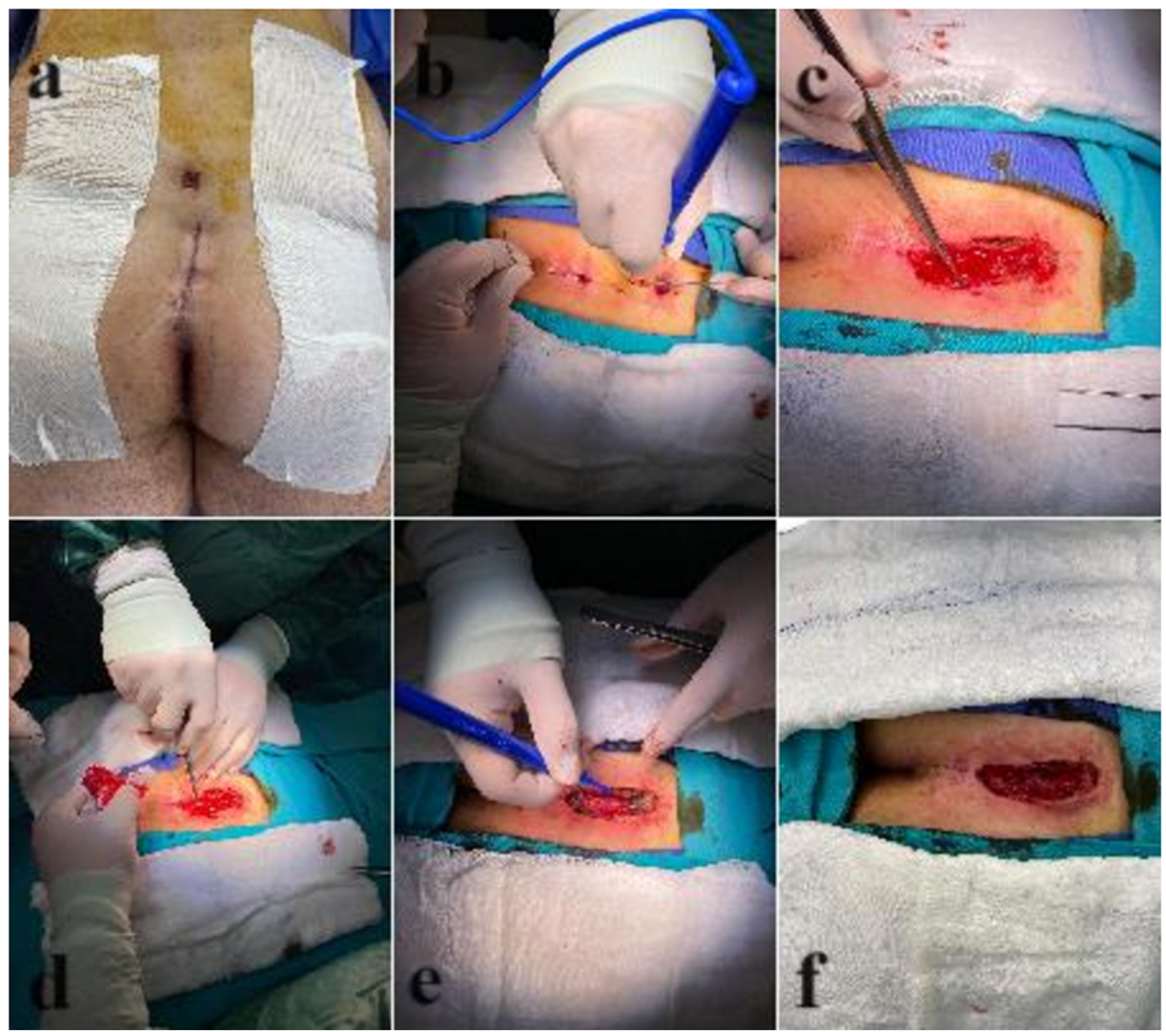
Step-by-step description of the technique. **(a)** Wound after the patient is positioned and the surgical site is shaved. **(b)** The sinus tract is explored with a probe, and then unroofing is performed over the probe. **(c)** Hair can be seen in the opened wound. **(d)** The debris in the wound is removed by curettage. **(e)** Hemostasis is achieved. **(f)** Final wound.

The sinus was probed and examined. The length and direction of the sinus were identified, and the sinus and extensions, if present, were unroofed by cutting directly with a diathermy over the probe. The hair and debris were cleaned, and the underlying epithelia were curetted and removed. Sites of bleeder were cauterized, and the wounds were packed with gauze (Figure 1).

### Postoperative care and follow-up

All patients were discharged within 24 h of the operation. None of the patients received perioperative antibiotic treatment. A family member or a friend of the patient was trained on how to change the wound dressing when nursing services were not available. Wound healing was evaluated by a team member either weekly by clinical visits or by asking the patient to send a photograph of the wound.

### Statistical analysis

Continuous variables are presented as the mean and standard deviation (SD) or median (minimum- maximum) depending on assumptions. Categorical variables are presented as the number and percentage of the total. Student's *t*-test and Wilcoxon-Mann-Whitney *U*-test were used to compare continuous variables. *χ*2 and Fisher exact tests were used to compare categorical variables. The Kruskal Wallis test was used for comparison of multiple-group analyses with a *post hoc* Bonferroni multiple comparison test. Kaplan–Meier analysis was performed to estimate the overall recurrence-free probability. *P* values <0.05 were considered statistically significant. All analyses were performed with SPSS version 13.0 (SPSS Inc., Chicago, IL).

## Results

During the study period, 74 patients underwent unroofing for recurrent pilonidal disease. The mean age was 29.8 ± 10.6 years. Sixty-one (82.4%) of the patients were male. The previous operations before recurrence were excision + flap reconstruction, excision + primary closure, local excision + lay open, and abscess drainage (Table 1).

**Table 1:** Characteristics of the patients (*n* = 74).

Variables	
Age (years)	29.8 ± 10.6
Male, *n* (%)	61 (82.4%)
Hospital type, *n* (%)	
Private hospital	22 (29.7%)
University hospital	52 (70.3%)
Total number of the operations	2.2 ± 0.6
Previous operations, *n* (%)	
WLE^a^ + flap reconstruction	30 (40.5%)
WLE + primary closure	23 (31.1%)
WLE + lay open	15 (20.3%)
Abscess drainage	6 (8.1%)
Follow-up duration (months)	81.6 ± 49.0 (6–159)
Return to work (days)	5.9 ± 3.0 (2–20)
Healing time (weeks)	6.5 ± 2.6 (3–16)
Operation time (minutes)	22 ± 5.3 (13–31)

^a^ WLE, wide local excision.

The mean operation time was 22 ± 5.3 min. The mean follow-up period after unroofing was 81.6 ± 49 months, and the shortest follow-up period was 10 months. The average time to return to work time was 5.9 ± 3 days. The average recovery time was 6.5 ± 2.6 weeks.

**Figure 2:**
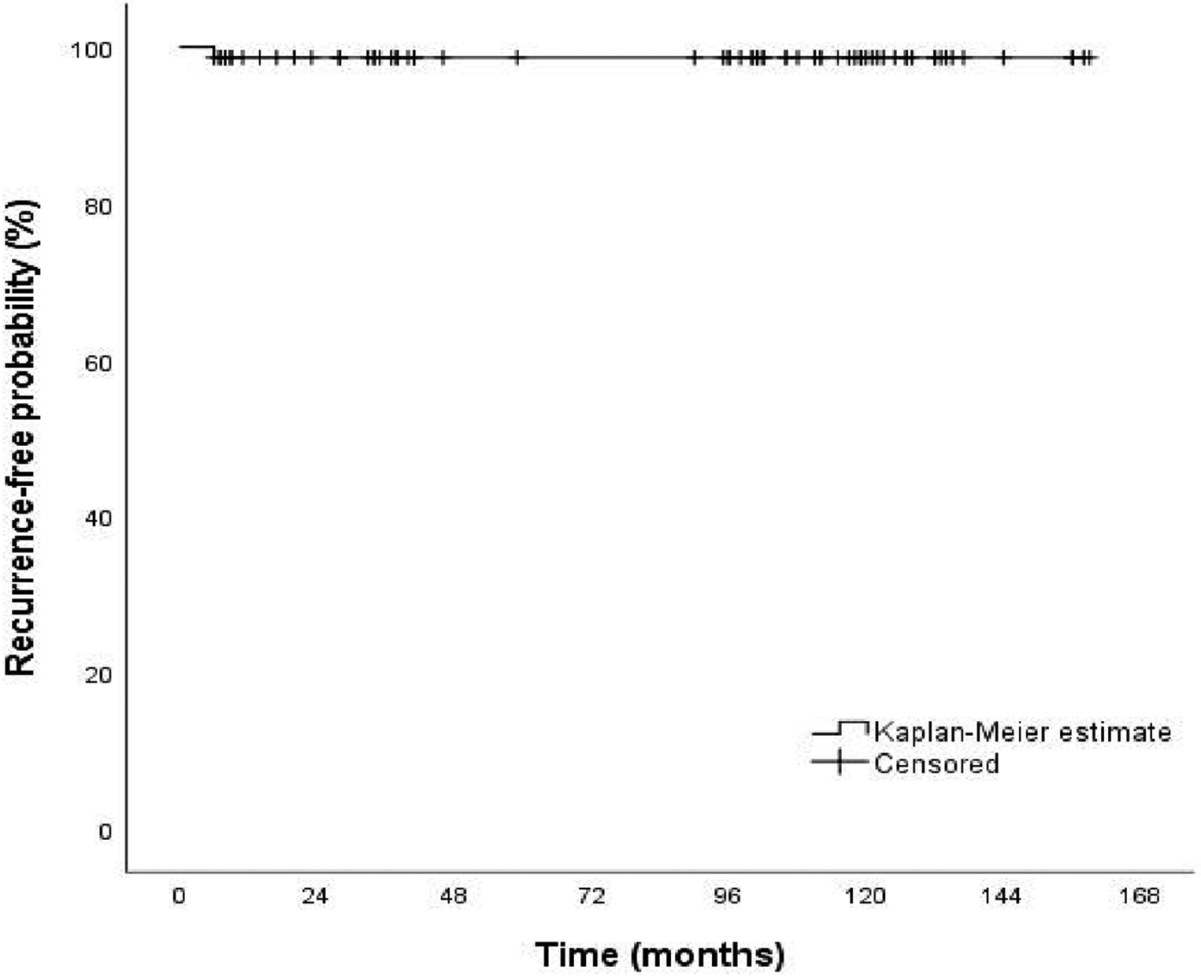
Kaplan Maier analysis for the recurrence risk after unroofing curettage (UC) operation.

Recurrence was observed only in one patient (1.3%) of the 74 patients. An UC operation was performed again for this case of recurrence at the 6th month of follow-up. According to Kaplan–Meier analysis, the recurrence-free rate of the patients at 60 and 120 months was 98.6% (Figure 2).

Additionally, the mean recurrence-free interval for participants undergoing UC for recurrent SPD was 156.9 months, with a 95% confidence interval (CI) of 152.9–160.9 months.

When the patients were compared according to the type of surgery they underwent before recurrence, no difference was found in terms of return to work or healing time (Table 2).

**Table 2:** Comparison of patients according to the type of previous operations.

	Flap reconstruction (*n* = 30)	Primary closure (*n* = 23)	Lay open (*n* = 15)	Abscess drainage (*n* = 6)	*p*
Return to work (days)	5.8 ± 2.2 5.5 (2–11)	7.1 ± 4.4 6 (3–20)	4.9 ± 1.8 5 (3–10)	4.5 ± 1.4 4 (3–7)	0.069
Healing time (weeks)	6.5 ± 2.4 6 (3–12)	7.0 ± 2.9 6 (4–15)	6.1 ± 3.1 6 (4–16)	5.7 ± 0.8 6 (4–6)	0.523

In total 61 patients had a single operation before UC; 13 had 2 or more operations. When evaluated according to the number of operations before recurrence, no difference was found in terms of return to work or healing time (Table 3).

**Table 3:** Comparison of patients according to the number of previous operations.

	Previous operations = 1 (*n* = 61)	Previous operations >1 (*n* = 13)	*p*
Return to work (days)	5.6 ± 2.6 5 (2–20)	7.3 ± 4.3 7 (3–20)	0.078
Healing time (weeks)	6.4 ± 2.3 6 (3–12)	7.3 ± 3.9 6 (4–16)	0.684

## Discussion

Treatment of pilonidal sinus disease should be simple, cause minimal pain, require a short hospital stay, allow for rapid return to work, cause no significant change in quality of life, have a low recurrence rate, and be cost-effective (3). Given these criteria, excisional methods are usually preferred over minimally invasive methods in patients with recurrent sacrococcygeal pilonidal disease. We recommend that the choice of retreatment should be chosen according to the type of recurrence. Although we are now more motivated to favor less invasive methods such as phenol and EPSiT, even for selected recurrent cases, in the present cohort, we preferred UC, which required very limited excision under local anesthesia, without removing the fibrotic back wall of the sinus to become a source for cells and other tissue components that help healing. As a consequence, the re-recurrence rate was very low (1.3%).

**Figure 3:**
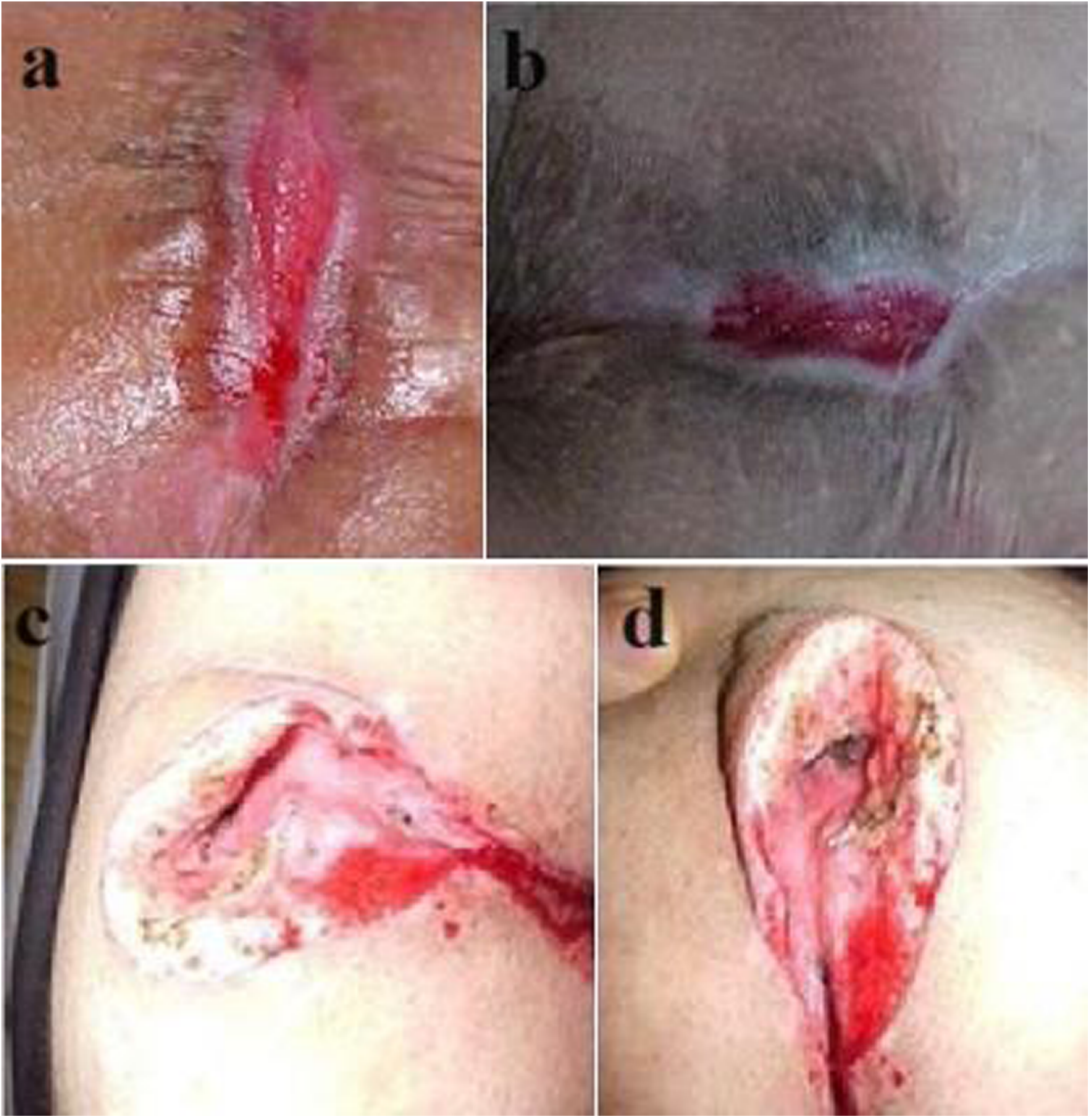
**(a** and **b)** Superficial recurrence, **(c** and **d)** Dehiscence of the wound.

**Figure 4:**
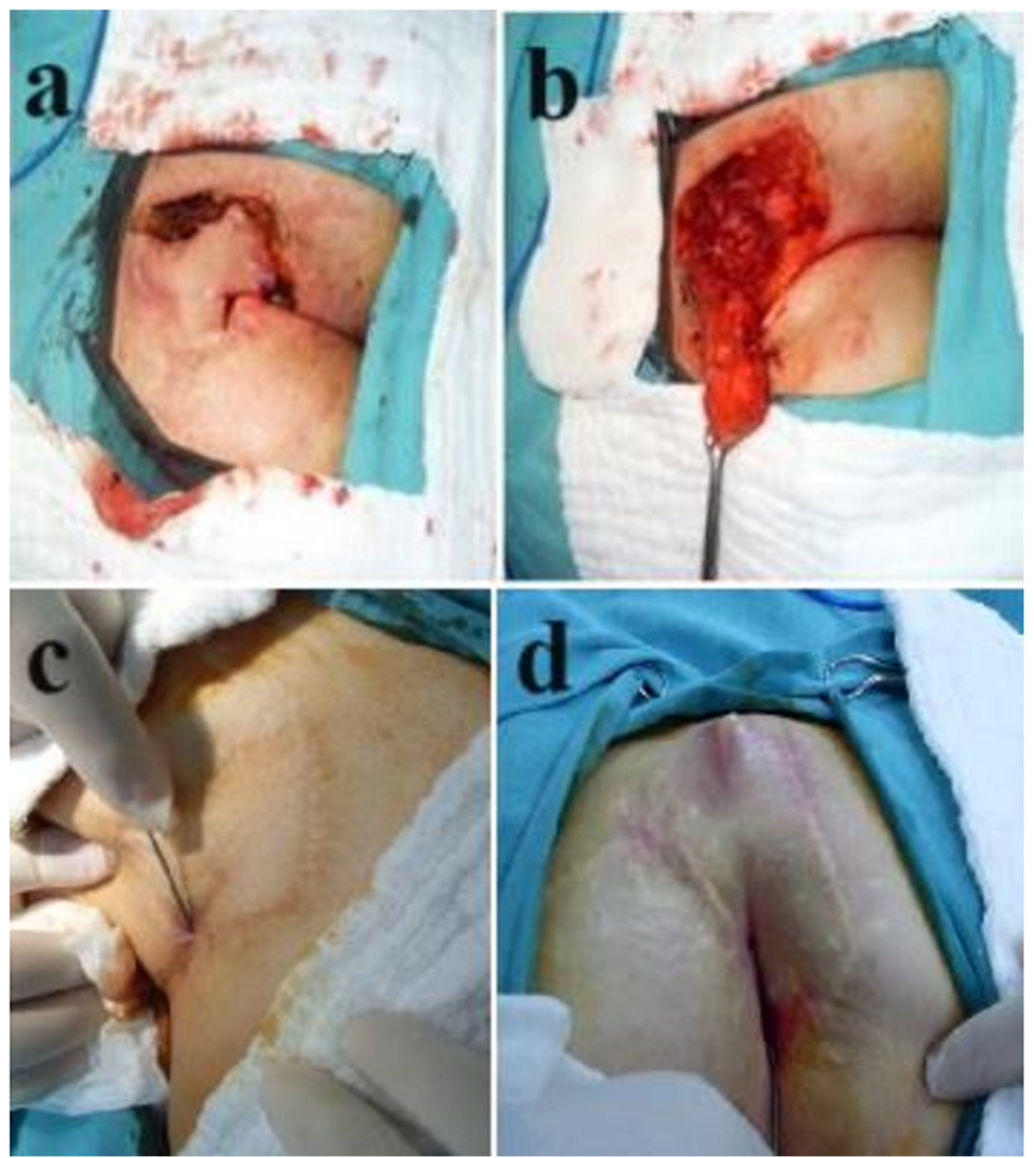
**(a and b)** Recurrence after healing problem with the flap, **(c and d)** Recurrence underneath an otherwise healed flap.

Varying rates of recurrence have been reported for pilonidal sinus disease surgery, depending on the technique, the geography (and thereby specific genetic mechanisms, health care settings and socioeconomic factors) and the length of follow-up (1). Overall, types of recurrence mostly depend on the previous surgical technique employed. The first group of recurrence is simple, superficial and easy to treat. This type of recurrence occurs mostly in low-BMI patients and especially after primary or secondary healing at the midline (Figure 3). The second type of recurrence occurs after attempts to elevate the midline groove to close the surgical defect. The problem is dehiscence of the wound and likely to be unrelated to the primary disease but obviously the way it was surgically treated. The third and fourth types of recurrences are related to rhomboid flaps. The third type is probably related to problems in lateral flap healing (Figure 4), and the fourth type is sinus formation underneath an otherwise healed flap.

For patients who present with abscesses, the incision + drainage method is not a preferred definitive choice for curative treatment. In fact, a recurrence rate of more than 35% can be seen at the 60-month follow-up. Wide local excision followed by repair with flaps or minimally invasive methods such as phenol application and EPSiT, recurrence rates vary between 0% and 5% after 12 months of follow-up (1, 2, 4). The longer the follow-up period is, such as 60 months, the higher the recurrence rate is, such as ranging between 2.7 and 19.8% (1). Previously published data mostly report 2%–6.2% recurrence rates in patients who undergo UC (1, 5–8); however, it can be as high as 13% at the 60-month follow-up, as demonstrated in the study by Doll et al. (1). Nevertheless, these studies involved heterogeneous groups that included both primary and recurrent cases, and little was reported about the types of recurrence. A classification of recurrences may help in understanding the extent of recurrence problems and is probably a better algorithm for treating recurrent disease. In the current study, although the patients were heterogeneous, the standard approach was UC, despite the disadvantages of secondary healing. Notably, a very low recurrence rate was detected even in an average follow-up period of 80 months.

Regarding treatment of pilonidal sinuses, return to work and healing time should be considered crucial factors. A study demonstrated that the median time to return to work for excision methods was 28 days for primary cases and 31 days after surgery for recurrence (9). Minimally invasive methods such as phenol, EPSiT and laser irradiation have a shorter average time to return to work (0–2.9 days) (2, 10–13) in most series. On the other hand, some studies report times to return to work between 3.2 and 4.3 days with the UC is, which are comparable to those of minimally invasive techniques (6, 7). Wound healing was much shorter with the UC method than with wide local excision. While healing time for WLE ranged from 10 to 160 days in various studies even if closure was performed after excision, the UC healing time was between 21 and 72 days (3, 5, 8, 14–17). Although short healing times are expected for methods such as phenol, EPSiT, and laser due to the nature of these minimally invasive methods, healing times were reported between 16 and 47 days in several studies (3, 10–13). Moreover, in a meta-analysis of pooled UC data, the time to return to work was reported to be 8.47 days (5). In the present group of patients, the average time to return to work was 5 days, and this variance is likely to be related to the type of social life, quality of life criteria, type of health insurance and whether sick leave is covered, and socioeconomic impact, which can motivate return to work.

The most important advantages of this study were less variance among surgeons, a long follow-up time and a sufficient number of patients for a recurrent pilonidal disease cohort. However, the limitation of this study was its retrospective design. Another limitation is that during the follow-up of these patients, recurrence was tracked as the main outcome, while other complications such as surgical site infections were not monitored or recorded. As we mentioned, the inclusion of patients with different recurrence types in this study introduces heterogeneity, which is a recognized limitation of the study. Although most patients presenting with recurrent pilonidal sinus during this study underwent UC surgery, a smaller number of patients with recurrent disease were treated with wide local excision, flap reconstruction, phenol treatment, EPSiT, and laser ablation. Unfortunately, since the patients who received UC treatment were not consecutively selected, there is a potential selection bias in this study.

In conclusion, there are various treatment methods for pilonidal sinus disease, and their evolution will continue for at least the short term. Patients and surgeons prefer a surgical strategy that is easy, quick and comfortable with an earlier return to work and social life as well as lower recurrence rates. The UC method somehow meets these criteria and will undeniably continue to be one of the treatment methods, at least in a selected group of recurrent patients. Further studies are recommended to classify recurrence and determine the effectiveness of UC in various patient subsets, which can be performed on an outpatient basis and is a lower cost technique; thus, it should be considered the first choice for treatment in recurrent cases.

## Data Availability

The datasets presented in this study can be found in online repositories. The names of the repository/repositories and accession number(s) can be found below: the datasets generated and/or analysed during the current study are not publicly available due confidentiality of patient data but are available from the corresponding author on reasonable request.
